# Non-Polar and Complementary Resistive Switching Characteristics in Graphene Oxide devices with Gold Nanoparticles: Diverse Approach for Device Fabrication

**DOI:** 10.1038/s41598-019-51538-6

**Published:** 2019-10-22

**Authors:** Geetika Khurana, Nitu Kumar, Manish Chhowalla, James F. Scott, Ram S. Katiyar

**Affiliations:** 1 Department of Physics, University of Puerto Rico, San Juan, Puerto Rico USA; 2 Department of Materials Science & Metallurgy, University of Cambridge, Cambridge, UK; 3 Department of Chemistry and Physics, University of St Andrews, St Andrews, UK; 4 Present Address: Western Digital Technologies, Inc. (SanDisk India Device Design Centre), Bangalore, India

**Keywords:** Materials science, Materials for devices, Information storage

## Abstract

Downscaling limitations and limited write/erase cycles in conventional charge-storage based non-volatile memories stimulate the development of emerging memory devices having enhanced performance. Resistive random-access memory (RRAM) devices are recognized as the next-generation memory devices for employment in artificial intelligence and neuromorphic computing, due to their smallest cell size, high write/erase speed and endurance. Unipolar and bipolar resistive switching characteristics in graphene oxide (GO) have been extensively studied in recent years, whereas the study of non-polar and complementary switching is scarce. Here we fabricated GO-based RRAM devices with gold nanoparticles (Au Nps). Diverse types of switching behavior are observed by changing the processing methods and device geometry. Tri-layer GO-based devices illustrated non-polar resistive switching, which is a combination of unipolar and bipolar switching. Five-layer GO-based devices depicted complementary resistive switching having the lowest current values ~12 µA; and this structure is capable of resolving the sneak path issue. Both devices show good retention and endurance performance. Au Nps in tri-layer devices assisted the conducting path, whereas in five-layer devices, Au Nps layer worked as common electrodes between co-joined cells. These GO-based devices with Au Nps comprising different configuration are vital for practical applications of emerging non-volatile resistive memories.

## Introduction

RRAM has gained great interest and development as the next generation of emerging non-volatile memory due to its promising performance and potential, including simple structures, scalability, fast speed, excellent endurance, low power consumption and compatibility with CMOS Technology^1–6^. Apart from its crucial memory applications, RRAM devices have exciting future possibilities to be employed in artificial intelligence and neuromorphic computing^7–10^. RRAMs are based on the voltage dependent resistance change of an oxide material sandwiched between two metal electrodes, comprising a Metal-Insulator-Metal (MIM) structure^11–13^. With the application of an appropriate voltage, the RRAM cell can be switched between a high resistance state (HRS) and a low resistance state (LRS). Depending upon the polarity, resistive switching can be classified in two types: Unipolar and Bipolar. Unipolar switching depends upon the amplitude of the voltage applied and not on the polarity of the applied voltage, whereas for bipolar switching, one polarity is used to switch from HRS to LRS, and the opposite polarity is used to switch back into HRS. Nonpolar resistive switching also exists, which is a coexistence of unipolar and bipolar resistive switching characteristics^14^. In nonpolar switching, the switching from LRS to HRS (the reset process) and from HRS to LRS (the set process) is done by the application of either positive or negative voltage. Bipolar switching has faster switching speed, better uniformity, and lower operational power^15^, while unipolar switching exhibits higher R_ON_/R_OFF_ ratio and high density integration^16^. Hence nonpolar resistive memories, which exhibit both unipolar and bipolar switching behavior, have been considered advantageous and can be seen as potential candidates for extended future applications in memory devices^17^. An ideal nonpolar switching device encompasses a total of four possible switching behaviors, of which two are for bipolar and the other two for unipolar switching modes^18^.

Furthermore, RRAM devices show great potential to be integrated into the high-density crossbar arrays because of its simple two-terminal structure and minimum cell size^19–22^. The crossbar architecture exhibits the commendable feature of an ideal 4F^2^ cell size that is promising for high density RRAM integration, establishing it as an emerging non-volatile memory; but the crossbar architecture has the major issue of sneak path current^23^. Sneak path is the route taken up by the current through de-selected cells and is detrimental to the operation of the memory cell, as it may vary the charge stored in the cell, which results in incorrect reading of the memory cell status^23^. Currently, to resolve the problem of sneak path, passive devices such as diodes or transistors are connected to the memristor; or alternatively, the complementary resistive switching (CRS), first proposed by Waser *et al*.^24,25^, is a solution to the sneak path current issue in crossbar arrays. CRS is composed of two RRAM cells connected in anti-serial fashion, separated by a common metal electrode. In this design one of the cells in CRS will always be in HRS or OFF state at low voltage, so the problem of sneak current is reduced effectively without the need of selector elements^26,27^. In this architecture excellent scalability is also possible by producing the smallest theoretical cell size, i.e., 4F^2/n^, where F is the minimum feature size and n is the layer number when 3-dimensional stacking is used^28–30^. Opting for resistive switching material, graphene was preferred due to its excellent properties exploited for logic devices. But as with the oxide/insulating switching matrix requirement for RRAMs, GO was chosen as the switching material, so that logic and memories can be implemented on the same platform. Recent studies demonstrated GO as a promising material for RRAM applications, owing to its easy processing, high flexibility, and excellent resistive-switching performance^31–35^. GO-based RRAM devices have shown excellent bipolar resistive switching characteristics^36–40^ and high on/off ratio, but the nonpolar resistive switching (NRS) and CRS in GO has not been studied much. Therefore, in this study we have presented the NRS and CRS results of GO-based devices in two different structures. It is a matter of device geometry which resulted in two different resistive behaviors. The point of attraction in our study lies in the simple fabrication process of the devices in two different structures, which was otherwise need high-tech processing techniques to fabricate the CRS architectures. Gold nanoparticles (Au Nps) used in these devices, mixed with GO matrix to fabricate GOAu thin film by spin coating for tri-layer ITO/GOAu/Al devices, resulted the NRS behavior in one structure (device A); a thin film of Au Nps was used as the common electrode separating the two RRAM cells in five-layer ITO/GO/Au NPs/Al_2_O_3_/Al devices showing CRS behavior in the other structure (device B).

## Results/Discussion

The schematics of device (A) and (B) are shown in Supporting Information (Fig. S1a–f). Device (A) has three-layer structure as ITO/GOAu/Al, with bottom electrode ITO/glass substrates, GOAu, the oxide/switching matrix, and aluminum (Al) as top electrodes. Whereas device B consists of five layers ITO/GO/Au NPs/Al_2_O_3_/Al with bottom electrode is ITO/glass substrate, the second layer is of GO; the middle or common electrode is of Au Nps; the fourth layer is aluminum oxide (Al_2_O_3_); and lastly the top electrode is Al. This five-layered device structure can be considered as connecting two memory cell elements in anti-serial fashion (cell-I and cell-II, as shown in Supporting Information Fig. S1f). Based on the difference in the assembled structure of the two devices A and B, we observed two different types of resistive switching characteristics. In device A nonpolar resistive switching was observed, which is a combination of bipolar and unipolar switching, whereas CRS switching was observed in device B. The resistive switching characteristics were measured by using the DC voltage sweep in top-bottom configuration in both the devices at room temperature.

### Device A

**Figure 1:**
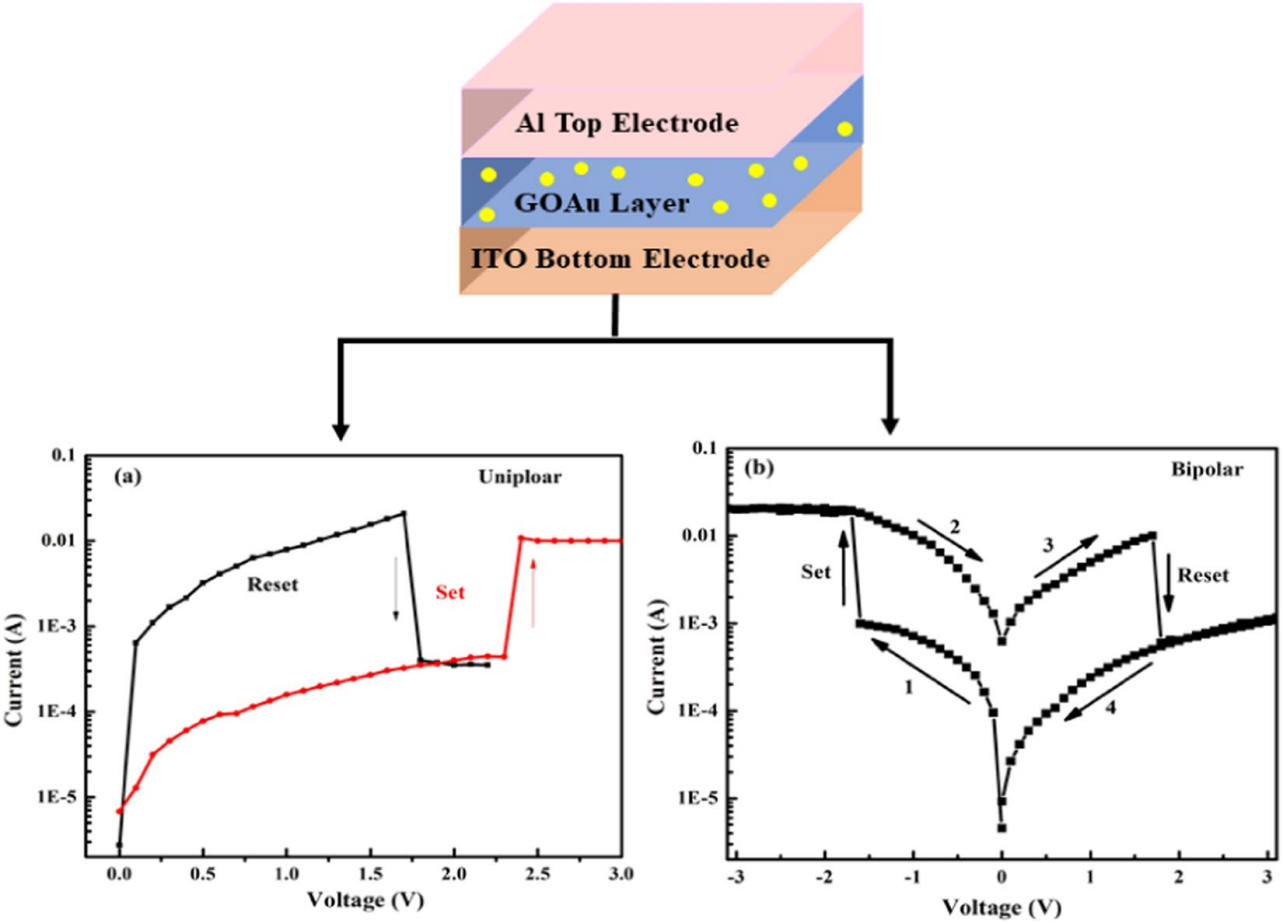
Typical I–V characteristics in device A for different switching modes, (**a**) unipolar switching; (**b**) bipolar switching.

The schematic and typical current-voltage characteristics for device A are shown in Fig. 1. The device exhibits nonpolar resistive switching behavior, which is the coexistence of unipolar (Fig. 1a) and the bipolar (Fig. 1b) switching behaviors. When the positive bias was applied, the Set process, which is the transition of the state from high resistance to low resistance, was observed at 2.3 V and the current increased sharply while re-sweeping, the positive voltage resulted in Reset process at 1.7 V, which is the switching of state from low resistance to high resistance; current was found to be decreased abruptly.

This is positive unipolar switching, as shown in Fig. 1a. Similarly, when the negative bias was applied, Set was achieved and Reset was obtained by re-sweeping the negative voltage, which is negative unipolar switching (not shown here). Furthermore, the bipolar switching was attained by continue sweeping the voltages from negative to positive or vice versa. Thus two types of bipolar switching were also observed, as Set (−1.6 V)/Reset was achieved by one type of voltage polarity, and Reset (1.7 V)/Set was obtained by reversing the polarity of the voltage, as shown in Fig. 1b. Hence, all the four types of behavior, two for unipolar and two for bipolar switching, are occurring in this device. The graphs shown in Fig. 1a,b clearly depict the switching behavior occurring in device A, which is a typical nonpolar characteristic of the device.

### Conduction mechanism

**Figure 2:**
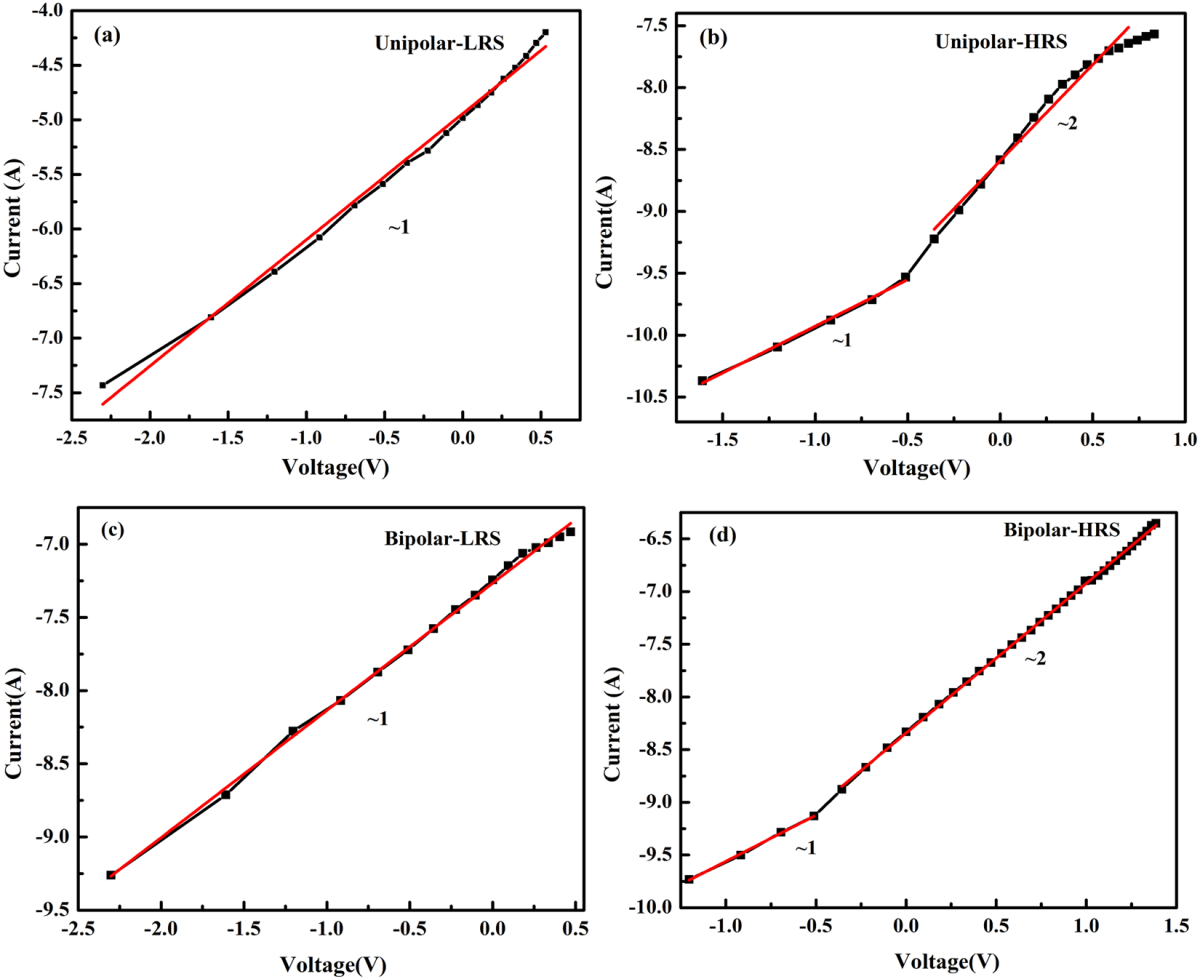
Typical I–V characteristics in log-log scale for unipolar (**a**) LRS (**b**) HRS region, and bipolar switching (**c**) LRS (**d**) HRS region.

To understand the prevailing conduction mechanism in the device in unipolar and bipolar behavior, the I–V plots were redrawn in log-log scale as shown in Fig. 2a–d respectively. In unipolar switching (Fig. 2a) in LRS region the I–V slope was ~1, showing linear dependence between I and need not be ohmic, since this behavior can be elucidated by Schottky conduction in Simmons limit of short electron mean free path, whereas in the HRS region (Fig. 2b), the lower voltage region has slope ~1, while the I–V slope in high voltage region was found to be ~2, showing space charge limited conduction (SCLC)^41^, where the current varies as a square of applied voltage according to the following equation I(V) = αV + βV^2^. In HRS high voltage region there was some deviation from the fitted line also observed, which is due to Joule heating in this region^42^. In case of bipolar switching, the conduction mechanism of the device was very similar to the unipolar switching (as shown in Fig. 2c,d). Here also the device was showing slope ~1 in LRS which could be Simmons Schottky conduction, while in the high-voltage HRS region, the mechanism was found to be governed by SCLC.

### Switching mechanism

To further understand the mechanism of switching behavior, we performed X-ray photoelectron spectroscopy (XPS) measurements for device A (Supporting Information Fig. S4a,b) which clearly show the peaks corresponding to well-known functional groups present in GO sheets and the Au Nps at their respective binding energies. The graph shows that GO has different oxy- groups, signifying the presence of an abundance of oxygen in the matrix^43,44^. Although we have deposited top Al electrodes through thermal evaporation, due to the readily oxidizable nature of Al and the abundance of oxy-groups in GO, there occurs the formation of an interfacial aluminum oxide (Al_2_O_3_) layer at the interface of GOAu and Al electrodes^45^. This process will also induce oxygen vacancies in the region of GO. When positive voltage is applied on the top electrode, oxygen ions from the GOAu matrix start moving towards the interfacial layer of Al_2_O_3_, and oxygen vacancies get aligned to pave the path for a conducting channel to form through the GOAu matrix; also the Au Nps present in switching matrix work as tiny filaments, facilitating conduction channel formation and the device transit to LRS or Set state. However, on reversing the polarity of applied field, the back movement of oxygen ions into the matrix results in the rupture of the conducting path and the device goes to HRS or Reset state. This is the first type of bipolar switching where Set and Reset states were achieved by applying positive and negative voltages respectively to the top electrode. Similarly, the second type of bipolar switching in which Set and Reset states were achieved by applying negative and positive voltages respectively can be explained by equating the role of the ITO bottom electrode to that of the interfacial Al_2_O_3_ layer. It is noteworthy here that ITO also works as a reservoir for oxygen ions and oxygen vacancies^46^. Now in positive or negative unipolar switching, Set was achieved first by applying the positive or negative voltage in a manner similar to that in bipolar switching; however, when the second pulse of the same polarity was applied, excessive current flows, resulting in Joule heating, which can also be seen in Fig. 2b, where the I–V graphs deviate from the fitted line. This resulted in rupturing the conduction channel and having the device go to its Reset state.

### Retention and endurance characteristics

**Figure 3:**
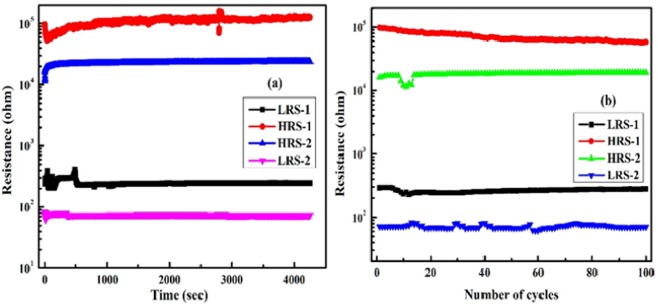
(**a**) Retention; (**b**) Endurance properties of device A in LRS-1 & HRS-1 for unipolar and LRS-2 & HRS-2 for bipolar switching.

Retention and endurance are the important properties of a memory device for its practical application. Retention behavior of the device was observed and is presented in Fig. 3a. LRS 1&2 and HRS 1&2 behavior in unipolar and bipolar switching was plotted as a function of time. The resistances were measured at a read voltage of 0.1 V for more than 4000 sec; not much degradation in the resistance values was observed in that period, which shows that device has good retention properties. Similarly, the LRS 1&2 and HRS 1&2 in unipolar and bipolar switching types were plotted versus number of cycles (Fig. 3b); the device maintains an almost constant ratio between LRS and HRS over 100 cycles. These test shows that device has good retention and endurance properties.

### Device B

**Figure 4:**
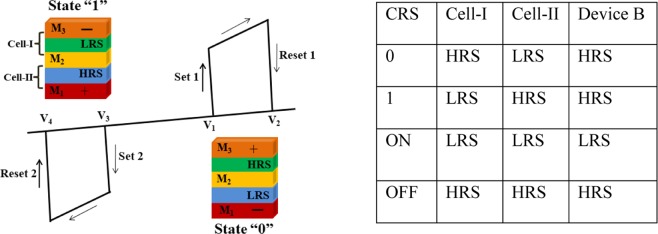
Schematic for typical I–V characteristics for a CRS device showing 0, 1 and ON states. Device structure with LRS and HRS in state 0 and state 1, having different electrodes and oxide matrices is also shown. The table presents the status of cell-I and cell-II corresponding to the status of CRS memory cell.

Owing to its different device geometry, we observed CRS switching behavior in device B, which is different from the NRS switching observed in device A. The ideal CRS graph and corresponding logic states are shown in Fig. 4, in which Set1, Reset1 and ON states are shown for one polarity of the voltage; and Set2, Reset2 and ON state are shown for the other voltage polarity. When the device goes from ON to Reset1, the state 0 is achieved, where cell-I is in HRS and cell-II is in LRS, whereas, the device reaches state 1 when it goes from ON to Reset2. Here the cell-I goes to LRS, and cell-II transits to HRS. When the voltage reaches V_1_, the current increases abruptly and both cells I & II switch to LRS; this state of CRS is the ON state. As the voltage keeps increasing and reaches voltage V_2_, the current drops suddenly via Reset1, and the CRS device goes to logic 0. When negative voltage is applied, the CRS device goes to ON state at voltage V_3_ and goes to state 1 at voltage V_4_. Until the voltage reaches V_1_ or V_3_, the current increases rapidly, and both the upper and lower cells are switched to LRS (Set-1 and Set-2). This state is the ON state and is generally used in the read operation. The voltage between V_1_ and V_2_ is to be applied to read the state of the CRS; hence the readout window can be defined as V_read_ = V_2_-V_1_. Therefore, the CRS cell has four different states, depending on the state of cell-I & cell-II: ON state (LRS/LRS), OFF state (HRS/HRS), state 0 (LRS/HRS), and state 1 (HRS/LRS). The bit information is stored in the two memory cells joined anti-serially (back-to-back), and the overall resistance of the CRS is dominated by the HRS. One of the cells will be in HRS; hence the CRS device will be always in HRS at low bias voltage, and is beneficial since it makes these devices free from the sneak current path problem.

As the CRS cell exhibits overall high resistance when storing the bit information, it effectively reduces the sneak current to the unselected cells. When a sufficient voltage is applied, both cells are turned ON to allow particular cell to be read. This concept provides an intriguing prospect for addressing the sneak path problem without introduction of any selector elements. Here the CRS state of the device is governed by the resistance state of cell-I and cell-II. When the device is in state 0, the cell-I is in HRS and cell-II is in LRS, and all the voltage drops across cell-I. The whole device then exhibits high resistance, whereas when the device is in state 1, the cell-I is in LRS and cell-II is in HRS, and all the voltage drops across cell-II. The whole device again exhibits the high-resistance state. The table given in Fig. 4 clearly shows the state of CRS when the states of cell-I and II are given with the resistance state of device B.

**Figure 5:**
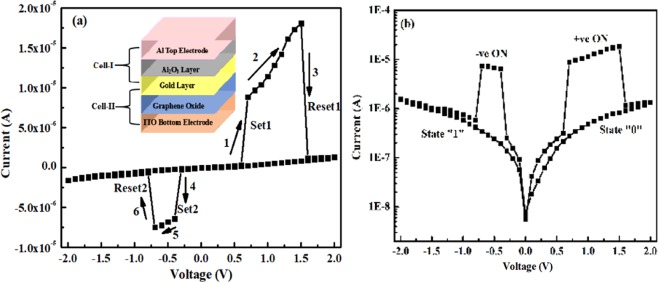
Typical I–V characteristics of device B in (**a**) linear scale (**b**) semi-log scale, showing various Set and Reset process, state 0, state 1 and ±ON states.

In this study device B is composed of two cells that are wired in series, having a separation by common metal electrode of Au Nps, as shown in the inset of Fig. 5a. The bottom cell-II here is composed of bottom electrode ITO, the switching matrix GO, and a common electrode of Au Nps, which also acts as the bottom electrode for upper cell-I, having Al_2_O_3_ as switching matrix and Al as the top electrode. Both the cells switch on and off according to the voltages applied and represent the states ON, OFF, 0 and 1. The current-voltage characteristics for the device are shown in Fig. 5a,b. Figure 5a is the I–V graph in linear scale, where sweeping directions are shown by arrows 1→6, along with Set and Reset process. Figure 5b is the I–V graph in semi-log scale, where CRS states of 0, 1 and ±ON are shown. Voltage was swept from 0 to ±2 V in both directions to observe the switching behavior of the device. When the positive voltage was increased on the top Al electrode, the current also started increasing gradually; at voltage V_1_(0.6 V)–i.e.,V_Set1_–there was a sudden jump in the current, and the device attained the ON state. As the applied voltage was increased further, a sudden fall in the current was observed at a voltage of 1.5 V, i.e. V_Reset1_, and the device went to state 0. As the applied voltage starts decreasing and goes negative, the device behaves in a manner similar to that of positive polarity, and we observe a sharp increase in current at −0.3 V i.e. V_Set2_ the device goes to ON state. Further sweeping of voltage resulted in a sharp fall in current at −0.7 V, i.e. V_Reset2_, and the device goes to state 1. The ON state windows were found to be ~0.9 V and 0.4 V, which are wide enough to read the data on that state. The maximum current values in the ON state are 12 µA at V_read_ of 1.1 V and 6.9 µA at V_read_ of −0.55 V. These current values are the lowest reported values in any CRS stack known to us until now. At ½V_read_ the corresponding current values were also found to be very low at 0.16 µA and 0.13 µA. The current ratios of ON states between V_read_ and ½V_read_ were found to be 75 to 53 (12 vs 0.16 µA, 6.9 vs 0.13 µA), which are much higher than the technology requirements (~10 μA) and are good for the operation of devices. The CRS behavior of the device was repeated continuously for several cycles, which is a promising characteristic of the device.

### Conduction mechanism

**Figure 6:**
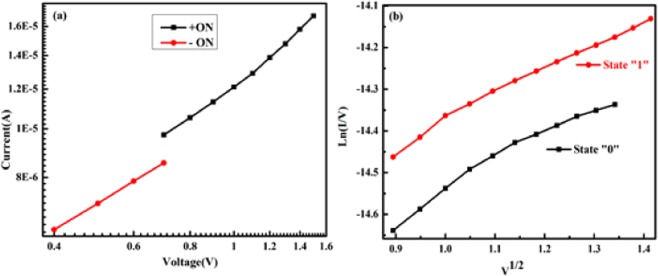
I–V graphs in log-log scale for (**a**) +ve/−ve ON state, (**b**) state 0 and state 1.

Graphs for I–V characteristics were redrawn to understand the conduction mechanism prevailing in the CRS device in different states. Figure 6a,b are the I–V plots for + ve/-ve ON state (LRS state) and state 0,1(HRS state). As can be seen from Fig. 6a, when the device is in state ON, the I–V plot is linear and the slope is ~1, showing that interface limited conduction is prevailing in the device. It is due to Schottky conduction in Simmons limit of short electron mean free path, whereas in Fig. 6(b) the I–V slope is non-linear and is exponential in V^1/2^. This nonlinear I–V behavior in HRS(state 0, 1) can be explained by Simmons’ modified Schottky Conduction mechanism given by equation:

1 $$J(E)=\alpha {T}^{3/2}E\mu {({m}^{\ast }/{m}_{0})}^{3/2}{\exp }[-(\phi /kT)+\beta {E}^{1/2}]$$

where 𝛼 is a constant; *T*, absolute temperature; E, applied electric field; *μ*, bulk mobility; *m*_0_, electron mass; *m*^∗^, effective mass of electron; *φ*, Schottky barrier height (SBH); *k*, the Boltzmann constant; *ε*_0_, the permittivity of free space; and *ε*, the optical dielectric constant. This form of the Schottky equation is appropriate whenever the mean free path of the electrons is less than the Schottky barrier width, which is generally true in oxides, where it is typically about one unit cell. Simmons’ equation has an ‘extra factor’ of *E* outside the exponent, as compared to original Schottky equation^47^.

### Switching mechanism

**Figure 7:**
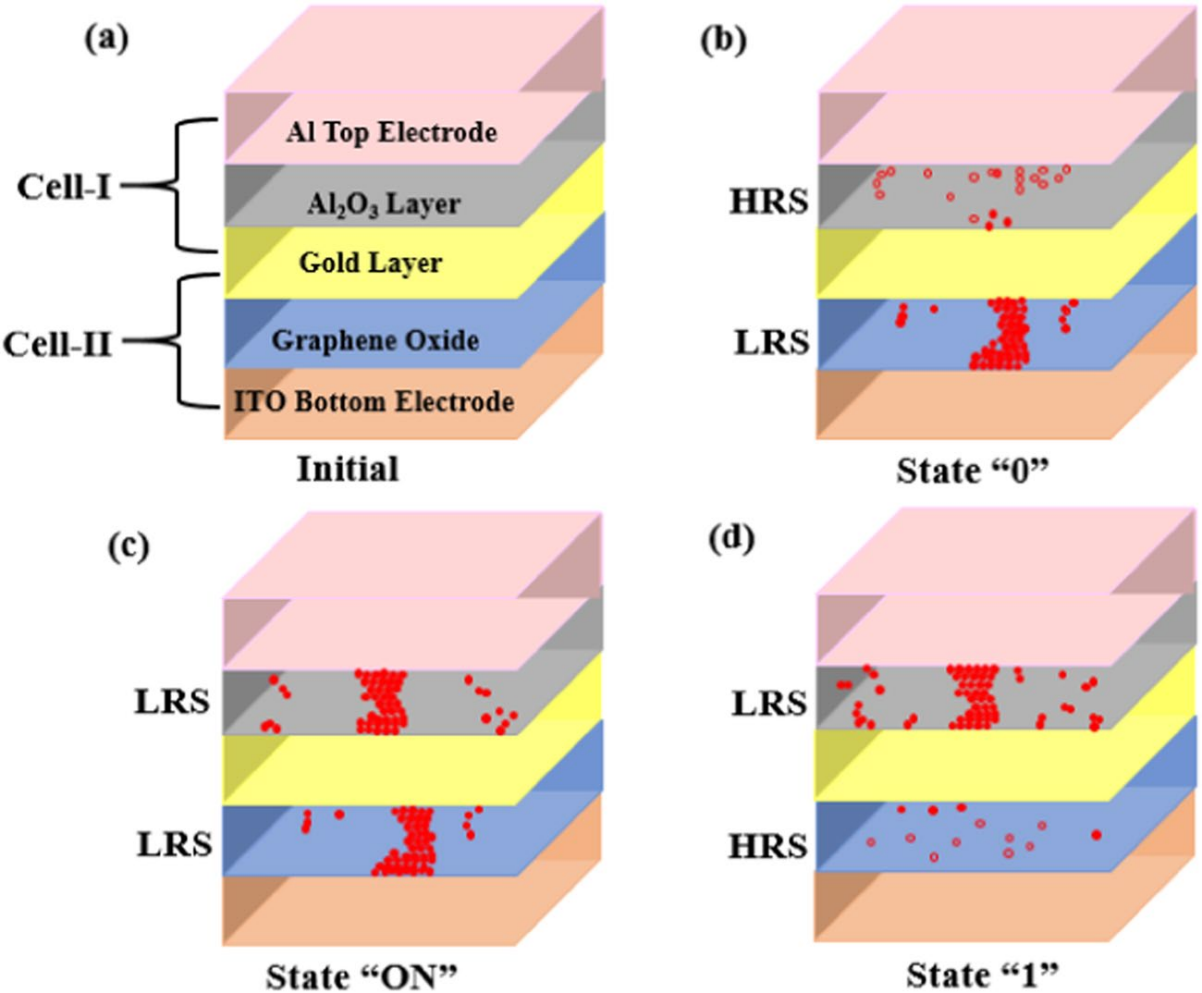
Schematic depicting switching process in device B: (**a**) initial state showing cell-I and II having five different layer; (**b**) state 0 showing breaking of filament in cell-I and formation of filament in cell-II; (**c**) ON state showing filament formation in both the cells; (**d**) state 1 showing conducting filament formation in cell-I.

The schematic of switching mechanism is shown in Fig. 7(a–d). Initially the device is in OFF state as both the cells (I & II) are in HRS. A complete cycle of forming voltage ~3 V was applied to Set the devices. As the positive voltage started increasing on top Al electrode and negative voltage on bottom ITO electrode, the cell–II containing GO as the switching matrix has a number of oxygen vacancies, which helps enable the formation of conducting path as a result, it goes to LRS. The cell-I is in HRS due to deficiency of oxygen, and charge trapping occurs due to the presence of Au electrode as the middle layer; the CRS device is in state 0. After a particular voltage of 0.6 V, the device turns to ON state, and both the cells are in LRS. The cell-I turns to LRS as it overcomes the trapping potential of Au Nps and cell-II was previously in LRS.

As the voltage kept on increasing the sudden fall in current was observed at 1.5 V, and the device attains state 0, whereas the cell-II remains in LRS and cell-I goes to HRS due to the breaking of the conduction path. At this point the device has maintained its state 0 as the voltage was reduced to zero, showing the non-volatile nature. Now if the polarity of voltage was reversed, i.e. negative voltage was applied to top Al electrode and positive voltage was applied to the bottom electrode ITO, the device was still in state 0, but at −0.3 V, the device again turns to ON state with both the cells in LRS. As the cell-II was in LRS and cell-I also switches to LRS due to the induction of oxygen vacancies from interfacial oxide layer and building the conductive path. Then with the increasing voltage, a sudden drop in the current was observed at −0.7 V and the device attained state 1 with cell-I in LRS and cell-II in HRS. Here cell-I goes to HRS due to the de-trapping of charges from Au Nps which increased the resistance inside the cell, breaking the path. Now the device will be in state 1 until the next ON state. Further, over a number of cycles of sweeping voltages from +ve to −ve the device was observed to switch from state 0 to state 1 and so on.

### Cumulative probability distribution

**Figure 8:**
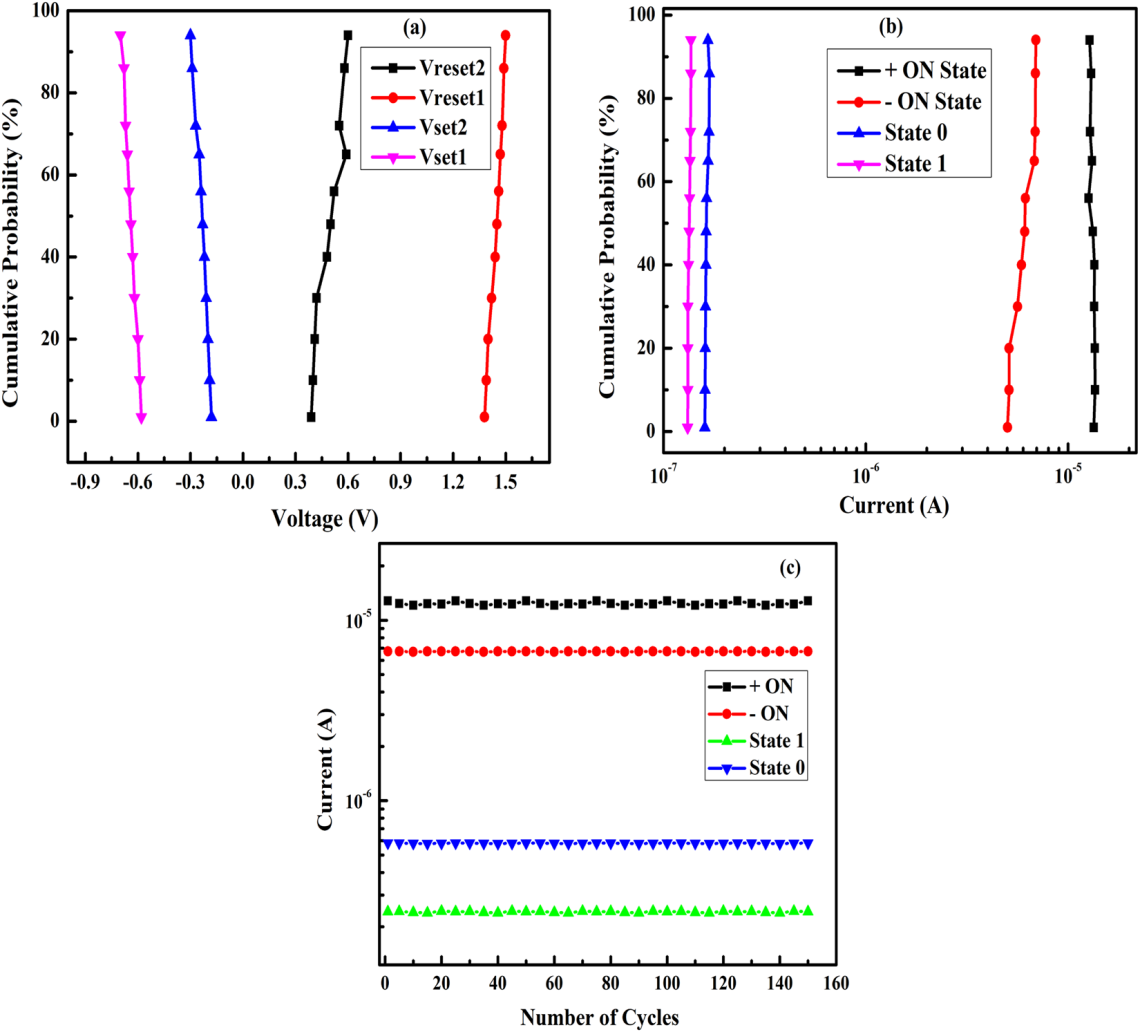
(**a**) Cumulative probability distribution of voltages as V_Set1_, V_Set2_, V_Reset1_ and V_Reset2_. (**b**) Cumulative probability distribution of currents in different states as state 0, state 1, +ve and −ve ON state. (**c**) Current values in state 0, state 1, ± ON state vs number of cycles showing endurance of device B.

Cumulative probability distribution of voltages and current values in state 0, state ON and state 1 are shown in Fig. 8a,b. The average values of Set1, Reset1, Set2 and Reset2 were found to be 0.5, 1.45, −0.25, −0.65 V respectively. Therefore, the negative and positive read merging are 0.95 V and 0.4 V respectively. The average values of current in positive and negative ON state, state 0 and state 1 were found to be 13, 6, 0.16 and 0.13 µA respectively. Therefore, the current ratios are approximately 81 and 46 for the positive and negative read voltages respectively, which is similar to the values shown in Fig. 5. The endurance properties of positive and negative ON states at read voltages of 1.1 and −0.55 V (V_read_) and for state 0 and 1 at 0.52 and −0.28 V (½V_read_) are shown in Fig. 8(c). The values are observed for 150 cycles which show no degradation in the current values, illustrating that the device has good endurance properties.

## Conclusions

In summary, the different resistive switching behaviors were observed in two devices owing to their different geometries. Device A is a three-layer structure having GO-Au as the switching matrix, and shows non-polar resistive switching, which includes two bipolar and two unipolar switching behaviors. In this device with the application of positive/negative voltage polarities, Set and Reset was achieved ~1.7 V along with good endurance of about 100 cycles and retention of ~10^4^ seconds. NRS behavior observed in this device is a good combination to have the faster speed and low power of bipolar switching and the high-density integration of unipolar switching, which was due to the presence of an abundance of oxy-groups and Au Nps in the switching matrix. Device B, which is a five-layer structure, shows complementary resistive switching behavior. This structure can be seen as two cells co-joined in serial fashion, where Al_2_O_3_ is the switching matrix in cell-I and GO in cell-II. This CRS device composed of a five-layer structure was fabricated using simple techniques of spin coating and thermal evaporation. The conduction channel was formed by oxygen vacancy alignment depending upon the application of voltage polarity and Set1, Reset1, Set2 and Reset2 were observed at low voltages (<1.5 V), and the corresponding storage states of 0 and 1 were achieved. As in any of the storage states, the CRS device is in HRS and is capable of suppressing the sneak path issue in crossbar architecture. Lowest values of current 12 µA at V_read_ of 1.1 V and 6.9 µA at V_read_ of −0.55 V were observed which is good for the write/erase process and one of the important factors for designing crossbar architecture.

## Methods

### Device fabrications

Graphene oxide was synthesized using modified Hummer’s method, as in our earlier study^37^. In brief, graphite powder (2 gm) was oxidized by an oxidizing agent (KMnO_4_, 7 g) in the presence of strong acid H_2_SO_4_ (50 mL) in an ice bath. KMnO_4_ powder was added very slowly to the solution of graphite powder and H_2_SO_4_ while maintaining the temperature below 5 °C. The mixture was then continuously stirred at room temperature for about 2 hours. Afterwards we added the distilled water to the solution. Then, hydrogen peroxide (H_2_O_2_, 30 wt%) was added slowly to the solution in order to facilitate the dissolution of any un-reacted KMnO_4_. Then the solution was centrifuged, and the slurry obtained was repeatedly washed with distilled water until pH was neutral. Finally, it was sonicated in distilled water to get the GO suspension having concentration 1 mg/ml. For the fabrication of device-A, commercially available gold nanoparticles were added to GO, and this solution was used for fabricating GO-Au thin films by spin coating onto ITO/glass substrates. Top electrodes of aluminum (Al) metal were deposited at high vacuum pressure by thermal evaporation technique using a shadow mask of diameter 200 µm onto the GO-Au film. Thus, the device structure formed was ITO/GOAu/Al. For the fabrication of device B, firstly the GO solution was spin-coated on ITO/glass substrates, and the films were dried in ambient atmosphere. Then the GO films were dipped into the solution of Au Nps for 5 hrs at room temperature. Al_2_O_3_ grown at low pressure and top electrodes of aluminum (Al) metal were deposited at high vacuum pressure by thermal evaporation technique using a shadow mask of diameter 200 µm over the film. Thus, the device structure formed was ITO/GO/Au/Al_2_O_3_/Al. So, the final structure of the device A is 3-layers ITO/GO/Al structure and B is the 5-layers ITO/GO/Au/Al_2_O_3_/Al structure.

### Characterization

Structural characterizations of the GO and GO-Au thin films were done using X-Ray Diffraction (XRD) and Raman Spectroscopy measurements (Supporting Information Figs S2 and S3). Also, the X-ray photoelectron spectroscopy (XPS) was done to observe the elemental composition of the GOAu thin film (Supporting Information Fig. S4). The resistive switching characteristics were measured by applying voltage to the top Al electrode and the bottom ITO electrode using a Keithley 2401.

## Supplementary information

Supplementary Information
