# Supplementary material for: Non-Polar and Complementary Resistive Switching Characteristics in Graphene Oxide devices with Gold Nanoparticles: Diverse Approach for Device Fabrication
Source: Sci Rep. 2019 Oct 22;9:15103. doi: 10.1038/s41598-019-51538-6 (PMC6806005; doi:10.1038/s41598-019-51538-6)
Supplement: Supplementary file 1 — Supplementary Information [file 41598_2019_51538_MOESM1_ESM.pdf]

Supplementary Information

Non-Polar and Complementary Resistive Switching Characteristics in Graphene Oxide devices with Gold Nanoparticles: Diverse Approach for Device Fabrication

Geetika Khurana[1], Nitu Kumar[1,4], Manish Chhowalla[2], James F. Scott[3], and Ram S. Katiyar[1]

[1]Department of Physics, University of Puerto Rico, San Juan, Puerto Rico, USA.

[2]Department of Materials Science & Metallurgy, University of Cambridge, UK.

[3]Department of Chemistry and Physics, University of St Andrews, St Andrews, UK.

[4]Current Affiliation-Western Digital Technologies, Inc. (SanDisk India Device Design Centre), Bangalore, India

Supplementary Discussion S1

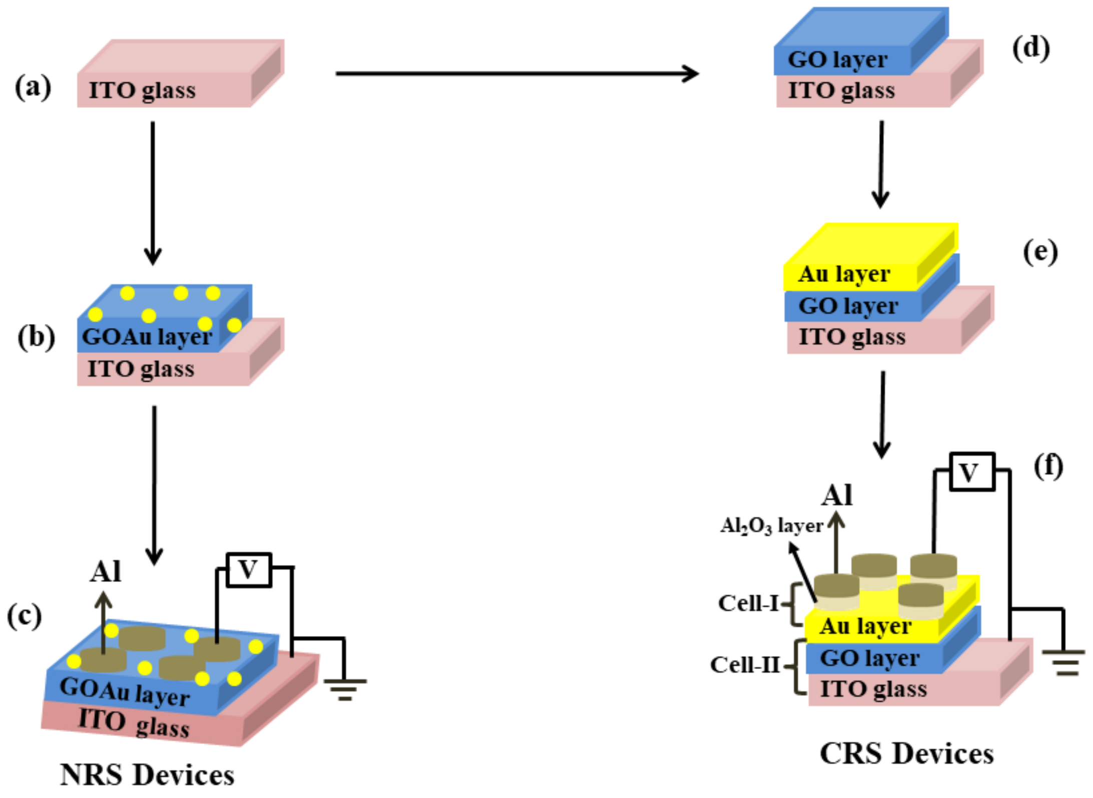

Figure S1.(a-f) Schematic for the various steps of device formation of device A and device B.

Figure S1 shows the step by step fabrication process for device A and B. a) ITO/glass substrate is used as the bottom electrode for both the devices, b) GO-Au layer deposited on ITO/glass by spin coating, c) Finally the Aluminum (Al) top electrodes are grown by thermal evaporation technique, using a stainless steel circular shadow mask having diameter 200 µm, which resulted in ITO/GOAu/Al structure exhibiting NRS devices, d) GO layer is deposited on ITO/glass substrate by spin coating, e) Au Nps layer was deposited by dipping it in Au Nps solution, and f) finally Al_2O_3 grown at low pressure and top electrodes of Al at high pressure by thermal evaporation, which resulted in ITO/GO/Au/Al_2O_3/Al structure for CRS devices.

Supplementary Discussion S2

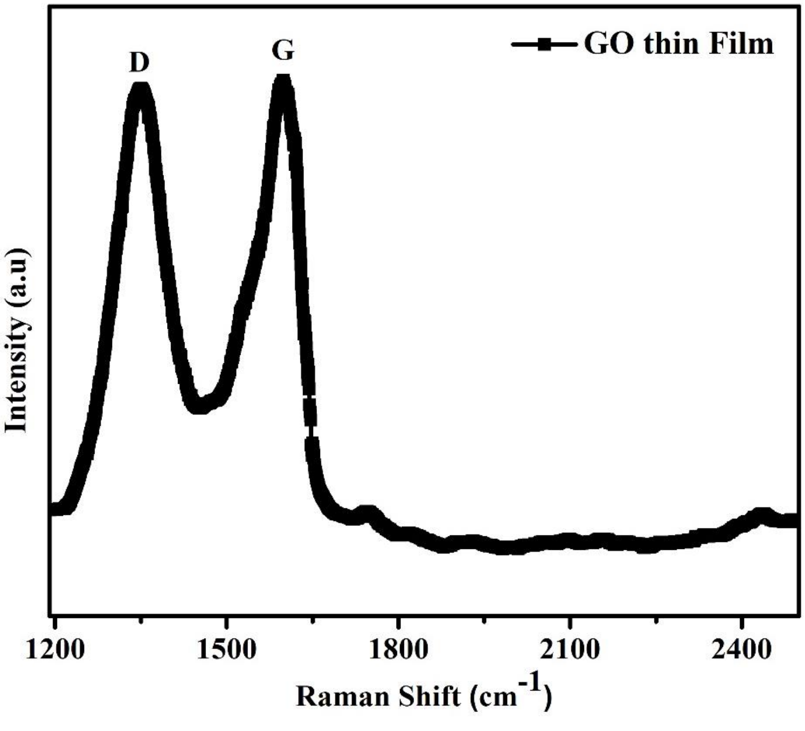

Figure S2. Raman spectra of GO Thin Film

Figure S2 shows the Raman spectra of GO thin film, which was carried out using a Horiba-Yobin T64000 micro-Raman system, having the 514.5 nm line of an argon ion laser as excitation source. The graph shows two characteristic peaks at 1359 cm^{-1} (D peak) and 1593 cm^{-1} (G peak). The D

peak signifies the presence of defect sites in the matrix of graphene oxide due to the disorder induced by sp^3 hybridization, whereas the G peak corresponds to the in-plane bond stretching motion of sp^2 carbon atoms.

Supplementary Discussion S3

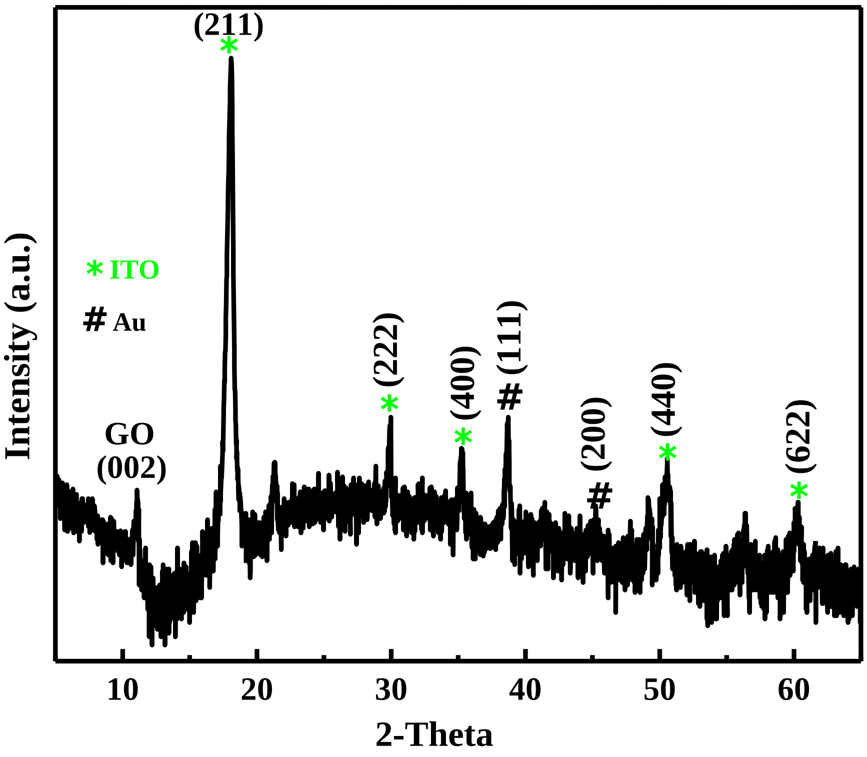

Figure S3. X-Ray Diffraction (XRD) spectra of GO-Au Thin Film

Figure S3 shows the XRD pattern of GO-Au thin film fabricated on ITO/glass substrate; and the observed peaks show the presence of GO, Au and ITO substrate. The peak (002) at 11.2 degrees is the characteristic peak for GO;and the peaks at 38.6, 45, and 66 degrees are for Au (#) corresponding to (111), (200), and (220) planes respectively. All other peaks shown by * are in accordance with the ITO/glass substrate.

Supplementary Discussion S4

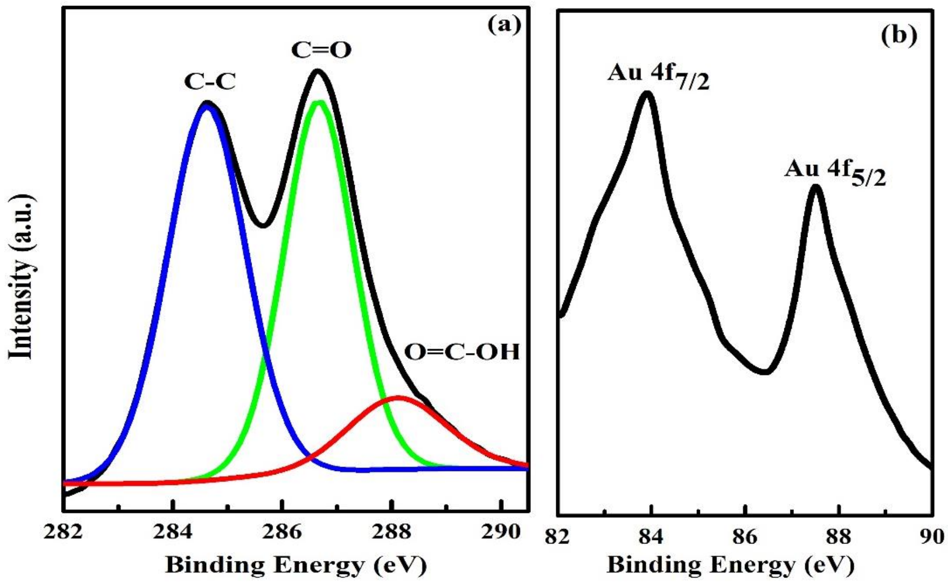

Figure S4. XPS spectra of GO-Au Thin Film

Figure S4 shows the XPS graphs of GO-Au film; the peaks corresponding to well-known functional groups present on GO sheets are clearly observed. Graph (b) shows the Au Nps at their respective binding energies. Figure S3(a) is for C1s spectra which has C-C, C-O and C=O peaks having corresponding binding energies of 284.6, 286.5, and 288.4 eV respectively. The C1s spectrum clearly indicates a higher intensity C=O peak in comparison to the C-C peak, which confirms the abundance of oxygen in the film. Figure (b) shows the Au $4f^{7/2}$ and Au $4f^{5/2}$ peaks having binding energies of 84 and 87.5 eV respectively, confirming the presence of Au Nps in the film.
